# Research on the Improvement of High-Temperature Performance of Asphalt by Waste Scallop Shell Powder

**DOI:** 10.3390/ma18050983

**Published:** 2025-02-24

**Authors:** Yi Chen, Xuejiao Cheng, Fangyuan Gong, Bingjie Fang, Yu Liu

**Affiliations:** 1 School of Highway, Chang’an University, South Erhuan Middle Section, Xi’an 710064, China; cy1127@zjvtit.edu.cn; 2 Road and Bridge School, Zhejiang Institute of Communications, No. 1515, Moganshan Road, Hangzhou 311112, China; 3 School of Civil and Transportation Engineering, Hebei University of Technology, 5340 Xiping Road, Beichen District, Tianjin 300401, China; 202031604003@stu.hebut.edu.cn (X.C.); 202131604018@stu.hebut.edu.cn (B.F.); 4 National Key Laboratory of Green and Long-Life Road Engineering in Extreme Environment (Changsha), Changsha University of Science & Technology, Changsha 410114, China

**Keywords:** pavement engineering, asphalt modifier, scallop shell powder, high temperature performance, modification mechanism

## Abstract

In order to utilize a large amount of waste scallop shells in road engineering and develop environmentally friendly and high-performance asphalt, the impact of scallop shell powder (SSP) on the high temperature performance of base asphalt and styrene-butadiene-styrene (SBS)-modified asphalt was evaluated in this study. The effects of SSP on the conventional properties of base asphalt and SBS-modified asphalt were investigated according to penetration, softening point, ductility, and rotational viscosity at 135 °C, and recommended dosing amounts were given, respectively. The high-temperature rheological properties of SSP-modified asphalt were evaluated according to temperature sweep and multiple stress creep and recovery (MSCR) tests. The modification mechanism of SSP-modified asphalt was analyzed by scanning electron microscopy (SEM) and Fourier transform infrared spectroscopy (FTIR). The results indicated that an appropriate amount of SSP can effectively improve the consistency and shear deformation resistance of base asphalt and SBS-modified asphalt but can also affect the ductility and fatigue cracking resistance. The recommended weight amounts of SSP in base asphalt and SBS-modified asphalt were 9% and 12%, respectively. Moreover, SSP can improve the thermal stability and resistance to permanent deformation of asphalt, but excessive SSP may weaken the improvement effect of the high-temperature rheological properties of asphalt. The SSP is evenly distributed and tightly combined with asphalt at an appropriate amount, and the modification process of asphalt mainly involves physical changes.

## 1. Introduction

In parallel with the advancement of the national economy and the improvement of residents’ living standards, the demand for production and consumption of aquatic products in the Chinese market is increasing. According to the Statistical Bulletin of National Fishery Economy in 2021, released by the Ministry of Agriculture and Rural Affairs, China’s production of shellfish products in 2023 reached a staggering 16.659 million tons [1]. Because 30–70% of inedible shells are generated during shellfish processing [2], the annual output of shellfish byproducts exceeds 10 million tons [3]. Shells are calcified structures formed by mollusks to protect the soft parts of the body and are mainly composed of 95% calcium carbonate and small amounts of organic materials such as proteins and polysaccharides [4]. Common types of shells include oyster shells, scallop shells, conch shells, etc. In traditional treatment methods, shells are often discarded or buried as common pollutants, which not only take up a substantial quantity of land resources, but also leads to the decomposition of salt attached to the shells by microorganisms, producing pungent gasses such as ammonia and hydrogen sulfide [5,6,7], causing serious impacts on the ecological environment and residents’ lives in coastal areas. Due to the development of the mechanical pressing method, the calcination method, and other methods, the large-scale production of shell powder has been achieved, significantly reducing the production cost. In recent years, the comprehensive utilization and disposal of shells as solid waste has attracted widespread attention. In addition to being used for craft, waste shells are also widely applied in various fields such as agriculture [8], water treatment [9], biomedicine [10], and construction engineering [11,12,13]. Bio-coatings made from ground shells have a porous, fibrous double helix structure that can effectively adsorb harmful substances such as formaldehyde, benzene, and ammonia and regulate air humidity [14]. The properties of shell powder as an inorganic material have also been used to improve the high-temperature crack resistance of high-strength concrete [15].

In recent years, due to their structural characteristics and chemical composition, shells have also gradually been applied in the field of road engineering, playing a role in improving soil-bearing capacity, partially replacing natural resources such as cement and aggregates, and serving as a modifier to improve the performance of asphalt materials [16,17,18,19]. The strength and stability of subgrade soil have a significant impact on the overall performance of roads, and shells can be used as reinforcing materials to improve the bearing capacity of the subgrade soil in unpaved roads in agricultural engineering. S. H. Rachmawati et al. found that adding shells to the soil can effectively increase the California bearing ratio (CBR) value of subgrade soil [20]. The following study showed that adding clam shells and cement to subgrade soil can improve the soil base’s shear resistance capability by enhancing the frictional angle within the subgrade soil [21]. To reduce the large carbon emissions caused by cement production, ground shell powder can be used as an environmentally friendly material to partially replace cement in the construction of semi-rigid base, rigid base, rigid pavement, and roadside facilities in road engineering. C. Chandrasiri et al. prepared a bio-cement using ground conch shells that showed similarities to Type I ordinary Portland cement (OPC) in terms of mechanical property and microstructure development [22]. Y. Han et al. used waste oyster shell powder and blast furnace slag as cement replacement alternatives in the preparation of sustainable concrete, which increased the compressive strength and reduced the carbon dioxide emissions of the mortar [23]. Shells of different sizes can be used as partial or complete substitutes for conventional coarse aggregates (such as basalt, limestone, gneiss, etc.) and fine aggregates (such as natural sand and artificial sand) in concrete, reducing the exploitation of natural resources [24,25]. Compared with conventional aggregates, shell aggregates have larger voids, higher water absorption, irregular surface and shape, and contain organic matter. M. Arabani et al. found that using shell powder as a filler to replace traditional minerals can improve the water stability, resistance to permanent deformation, and fatigue life of hot mix asphalt (HMA) [26]. D. H. Nguyen et al. used crushed seashells to replace 60% of the natural aggregates in conventional concrete and achieved good freeze–thaw resistance and drainage capacity, with durability meeting the requirements for low traffic load applications [27]. B. Peceno et al. used scallop and mussel shells with a particle size between 2 and 7 mm to completely replace natural coarse aggregates to prepare porous concrete for the construction of sound barriers in road engineering [28]. Compared to conventional porous concrete, the sound absorption coefficient was increased by 40%. N. N. Wurie et al. used non-calcined oyster shells as coarse aggregates and partially replaced cement with calcined oyster shell powder to strengthen and stabilize mine waste rock as road base material, achieving good strength performance and environmental benefits [29].

Insufficient thermal stability of asphalt will result in distresses of pavement such as shoving, corrugation, rutting, and so on. Therefore, the driving comfort and long-term performance of asphalt pavement will be affected. Studies have shown that calcium-based solid waste such as eggshells [30], shrimp and crab shells [31], and fish scales [32] can be used as modifiers to effectively improve the stiffness and thermal stability of asphalt. In order to improve the high temperature performance of asphalt materials and promote the large-scale utilization of waste shells, some scholars have conducted research on shells with similar material characteristics and elemental compositions as this type of solid waste. N. Nciri et al. [33] found that oyster shell powder increased the content of resins and decreased the content of aromatics in asphalt, thereby improving its rutting resistance. Wang et al. [34] found that oyster shell powder mainly improved the thermal stability and temperature sensitivity properties of asphalt through physical changes, leading to effective improvement of the high-temperature rheological and elastic recovery ability of asphalt. The effects of shell powder on the rheological and adhesion properties of base asphalt have been discussed [35,36], but the effect of scallop shell powder (SSP) on the high temperature performance of different types of asphalt still needs to be further researched.

It is important to investigate the effect of waste scallop shell powder (SSP) on asphalt properties to develop green pavement materials with good performance and reduce the negative impact on ecology and living environment, especially for projects such as island construction with a lack of traditional building materials and high freight-costs. Based on the observation of SSP microscopic morphology and infrared spectroscopy analysis, the regularity of conventional indicators (such as penetration, softening point, ductility, and rotational viscosity at 135 °C) with SSP content variation were studied to investigate the effect of SSP on the conventional properties of base asphalt and SBS-modified asphalt. The recommended SSP content in the two types of asphalt was proposed. Dynamic shear rheological tests (temperature sweep and MSCR test) were used to characterize and evaluate the effect of SSP on the high-temperature rheological properties of asphalt. The mechanism of SSP-modified asphalt was analyzed using SEM and FTIR tests.

## 2. Materials and Methods

### 2.1. Materials

#### 2.1.1. Asphalt

In this study, Shell 70# petroleum-based asphalt and SBS-modified asphalt were selected. The main technical indicators are shown in Table 1 and Table 2, which meet the technical requirements of JTG F40-2004 [37].

#### 2.1.2. Scallop Shell Powder

**Figure 1:**
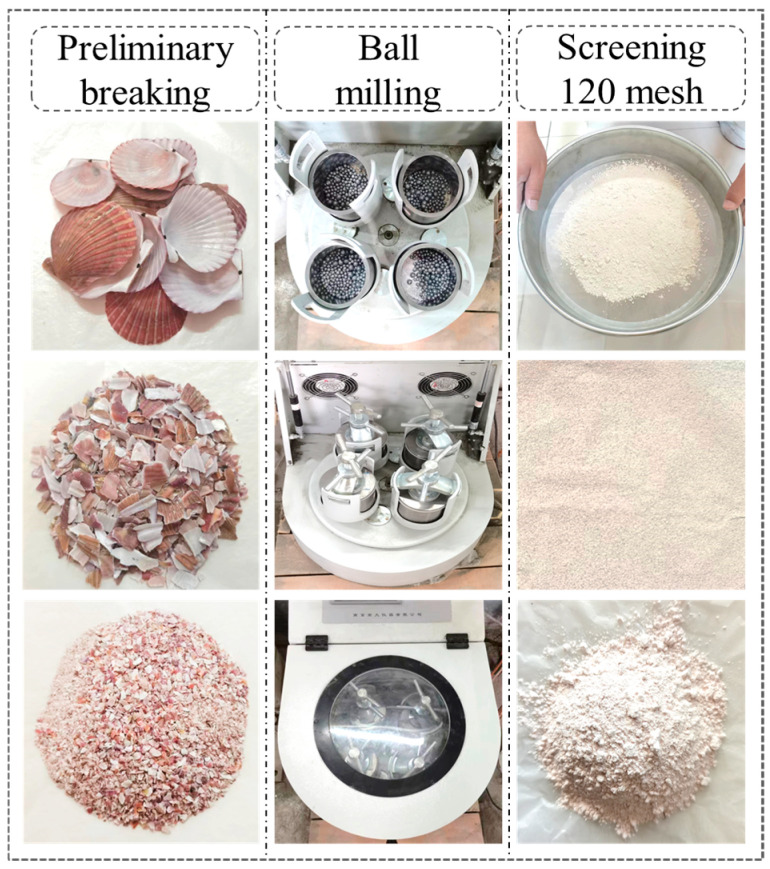
Preparation processes of SSP.

The scallop shells used in this study were obtained from Tianjin, China. The preparation process of SSP is shown in Figure 1. After being washed with tap water and dried naturally, the scallop shells were first crushed into smaller pieces by crushing equipment and then ground with a Nanda Instrument QM-3SP2 planetary ball mill (Nanjing, China). In each mill jar, 2, 20, and 100 stainless steel balls of large, medium, and small sizes were used, with the scallop shells and mill balls each occupying one-third of the space. The four mill jars rotated unidirectionally at 500 rpm and the planetary ball mill was stopped for 5 min for every 10 min of running time. The scallop shell powder smaller than 120 mesh was obtained by passing through a sieve of 0.125 mm after the running time reached 30 min. The unscreened large particles were ground in the next round along with the new scallop shells.

### 2.2. Preparation of SSP-Modified Asphalt

**Figure 2:**
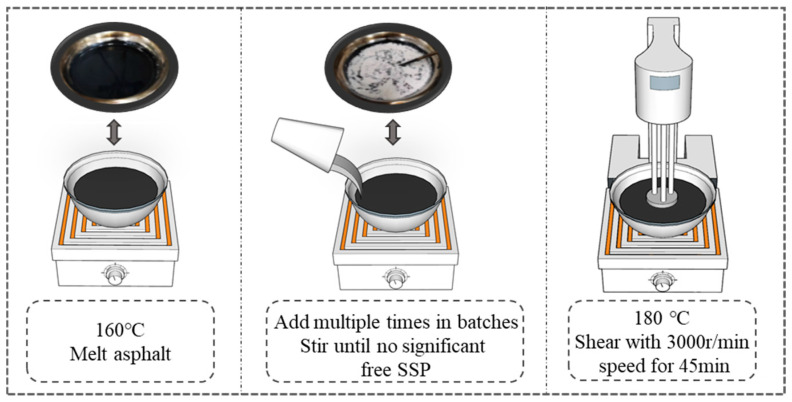
Preparation process of SSP-modified asphalt.

The preparation process of SSP-modified asphalt is designed as shown in Figure 2. Firstly, the asphalt was heated to 160 °C in a free-flowing state, which is beneficial for the combination and mixing of asphalt with SSP. During the stirring process, SSP was added to the asphalt in 3–4 batches, and then the asphalt was continuously stirred at low speed, until there was no visible free SSP, and gradually warmed up to 180 °C. Finally, the FJ300-SH high-speed dispersion homogenizer was used to shear and disperse the SSP in asphalt at a speed of 3000 r/min for 45 min. SSP with different weight contents (3%, 6%, 9%, 12%, and 15%) was added to the Shell 70# base asphalt and SBS-modified asphalt to prepare SSP-modified asphalt, and following the external addition method. For ease of reading, the testing groups were named BSSP3, BSSP6, BSSP9, BSSP12, BSSP15, SSSP3, SSSP6, SSSP9, SSSP12, and SSSP15.

### 2.3. Test Methods

#### 2.3.1. Conventional Properties Evaluation

The conventional performance evaluation indicators (penetration, softening point, ductility, and rotational viscosity at 135 °C) were tested according to the requirements in T0604, T0606, T0605, and T0625 of JTG E20-2011 [38], and the average values of 3, 2, 3, and 3 parallel dates were taken for obtaining reliable results, respectively. The penetration referred to the penetration depth of a 100 g standard needle into the asphalt sample at 25 °C within 5 s. The softening point was defined as the temperature at which a 3.5 g standard steel ball dropped to a specified depth from a metal ring filled with an asphalt sample. The ductility measured the length of asphalt in standard molds when stretched to the point of rupture at a speed of 5 cm/min at a temperature of 10 °C. The rotational viscosity was tested at a temperature of 135 °C with a Brookfield SC4-27 spindle [32].

#### 2.3.2. Dynamic Shear Rheological Tests

According to the requirements in T0628 of JTG E20-2011 [38], temperature sweep and MSCR tests were conducted utilizing an Anton Paar MCR 102 dynamic shear rheometer, 2 parallel samples were fabricated for each test. The temperature sweep test was carried out in strain-controlled mode with a strain of 12% and a frequency of 10 rad/s. In this research, the test temperature started from 46 °C and gradually increased by 6 °C. The complex shear modulus (G*) and phase angle (δ) are used as test indexes, and the test was stopped until the rutting factor G*/sinδ of the sample decreased to 1 kPa. The MSCR test on the RTFO-aged samples was conducted in creep-recovery-controlled mode at three test temperatures: 52 °C, 58 °C, and 64 °C. The samples were first measured for 20 loading cycles at a stress level of 0.1 kPa, followed by 10 loading cycles at a stress level of 3.2 kPa. The test indexes included recovery rate (R) and non-recoverable creep compliance (J_nr_).

#### 2.3.3. Scanning Electron Microscope Test

In order to observe the micro-morphology and surface structure of test materials, the samples of SSP and asphalt were imaged at 1000×, 5000×, 10,000×, and 500×, 1000×, 3000× magnifications, respectively, by a JEOL JSM-7800F field-emission SEM (Akishima, Tokyo, Japan). The powdered SSP was placed on a conductive tape and sprayed with gold for conductivity treatment directly. The asphalt sample should be rapidly cooled with liquid nitrogen first and then brittle fractured. The asphalt sample with exposed fresh fractures was conductively treated. 

#### 2.3.4. Fourier Transform Infrared Spectroscopy Test

FTIR tests were measured using a BRUKER TENSOR 27 Fourier infrared spectrometer (Billerica, MA, USA) with a wavenumber range of 4000–400 cm^−1^. The diluted asphalt sample was deposited onto a potassium bromide pellet and subsequently dried in an oven maintained at 50 °C prior to undergoing testing. The chemical analysis was carried out through the infrared spectrum of the sample.

## 3. Results and Discussion

### 3.1. Analysis of Scallop Shell Powder

**Figure 3:**
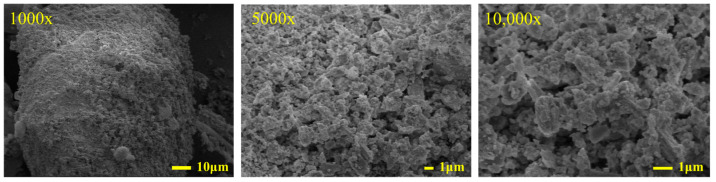
SEM micrographs of SSP at 1000×, 5000×, and 10,000× magnifications.

**Figure 4:**
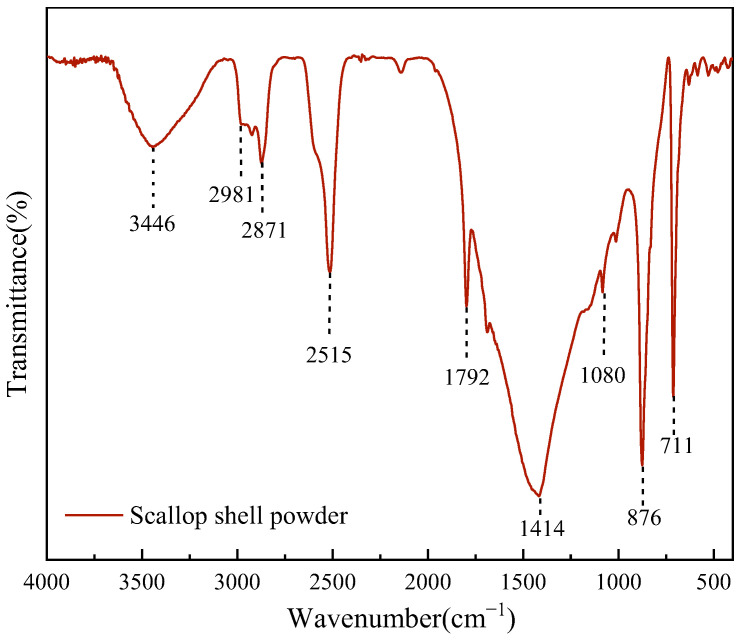
Fourier transform infrared spectra of SSP.

The microstructure of SSP was observed with a field-emission SEM, and the 1000×, 5000×, and 10,000× images of SSP were obtained, as shown in Figure 3. SSP and potassium bromide were made into a sample tablet by the HY-15 tablet press for infrared analysis, and Fourier transform infrared spectra of SSP was obtained as shown in Figure 4.

At 1000× magnification, the surface of SSP particles is rough and uneven. At 5000× magnification, the CaCO_3_ calcite-type crystal structures in SSP are varied in shape, uniformly distributed, and are interconnected to form a stable overall structure [39]. At 10,000× magnification, voids with a diameter of approximately 1 µm are observed between the CaCO_3_ crystal structures in SSP. The abundant void structures increase the specific surface area of SSP particles, thereby allowing SSP to fully contact with asphalt.

The absorption peaks at 1414 cm^−1^, 1082 cm^−1^, 876 cm^−1^, and 711 cm^−1^ of SSP are attributed to the antisymmetric stretching vibration, symmetric stretching vibration, out-of-plane bending vibration, and in-plane bending vibration of the CO_3_^2−^, respectively, indicating that the main inorganic phase in SSP was the CaCO_3_ of calcite-type crystal [40,41]. The absorption peaks at around 1792 cm^−1^ and 2515 cm^−1^ are produced by the stretching vibration of C=O and the antisymmetric stretching vibration of -SH-, respectively [42]. The absorption peak between 2871 cm^−1^ and 2981 cm^−1^ may be generated by -CH_3_- stretching vibration of proteins and amino acids in SSP [43]. The absorption peak of -OH may originate from the small amount of water content in SSP, and the absorption peaks of -SH- and N-H may be attributed to a small amount of chitin in SSP [44]. In summary, SSP mainly contains inorganic substances (CaCO_3_), as well as a small amount of protein and chitin.

### 3.2. Conventional Properties

The needle penetration, softening point, ductility, and rotational viscosity at 135 °C of base asphalt and SBS-modified asphalt with SSP contents of 3%, 6%, 9%, 12%, and 15% were tested. Needle penetration can reflect the relative viscosity and consistency of asphalt. Softening point is a key indicator for evaluating the temperature sensitivity of asphalt. Ductility can characterize the extensibility and fatigue cracking resistance of asphalt. The rotational viscosity at 135 °C reflects the ability of asphalt to resist shear deformation under external forces.

**Figure 5:**
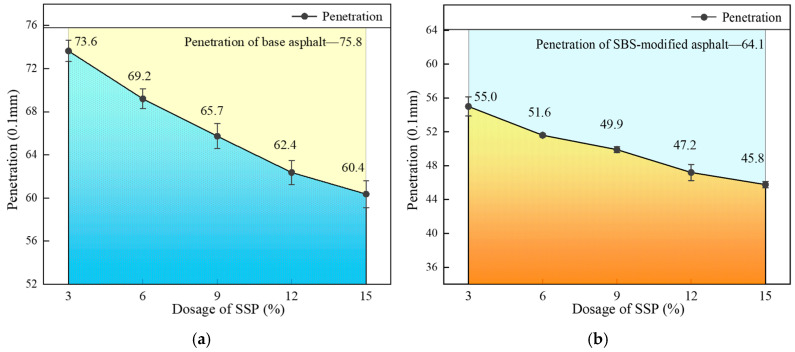
Penetration of base and SBS-modified asphalt with different SSP contents. (**a**) Base asphalt; (**b**) SBS-modified asphalt.

The effects of different SSP contents on the penetration of base asphalt and SBS-modified asphalt are shown in Figure 5a,b. Using the penetration values of base asphalt and SBS-modified asphalt as reference lines, the penetration of asphalt gradually decreases with the increase in SSP contents. The decreases in penetration of base asphalt with SSP contents of 3%, 6%, 9%, 12%, and 15% are 2.9%, 8.7%, 13.3%, 17.7%, and 20.3%, respectively, and the trend of decrease rate gradually slows down. The SBS-modified asphalt exhibits a similar pattern. It may be because the stronger SSP fills the internal structure of the asphalt, thereby increasing its viscosity and stiffness.

**Figure 6:**
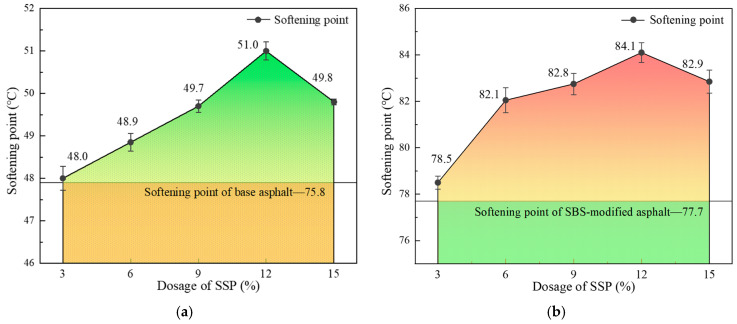
Softening point of base and SBS-modified asphalt with different SSP contents. (**a**) Base asphalt; (**b**) SBS-modified asphalt.

The test results of the softening point of base asphalt and SBS-modified asphalt with the change in SSP contents are shown in Figure 6a,b. Taking the softening point of base asphalt and SBS-modified asphalt as the baseline, the increase in softening point is relatively small with the SSP content of 3%, indicating that the temperature sensitivity of asphalt may be little enhanced with a small amount of SSP. As the content of SSP increases gradually to 12%, a significant increase in softening point appeared. And the softening point of asphalt began to decrease after the SSP content exceeded 12%. It is considered that the thermal stability of asphalt can be significantly improved with the increasing SSP content and the formation of a stable structure evenly distributed in the asphalt. However, the thermal stability of asphalt may be reduced by excessive SSP.

**Figure 7:**
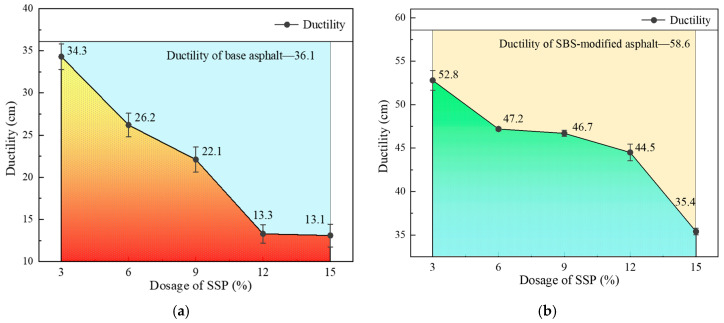
Ductility of base and SBS-modified asphalt with different SSP contents. (**a**) Base asphalt; (**b**) SBS-modified asphalt.

The effects of SSP on the ductility of base asphalt and SBS-modified asphalt are shown in Figure 7a,b. The ductility of asphalt decreases with the increase in the SSP content. A significant drop occurs in ductility after the SSP content exceeds a certain value, and this “diving point” for base asphalt and SBS-modified asphalt is 9% and 12%, respectively. At this point, the ductility of SSP-modified base asphalt cannot satisfy the requirements for 70# base asphalt in the JTG F40-2004 [37]. It is considered that SSP with high stiffness can become a weak point and affect the ductility and fatigue cracking resistance of asphalt. In addition, excessive SSP may become aggregated from dispersed particles and exacerbate stress concentration, while SBS-modified asphalt with a more stable structure can resist the impact of aggregated SSP particles on its ductility more effectively.

**Figure 8:**
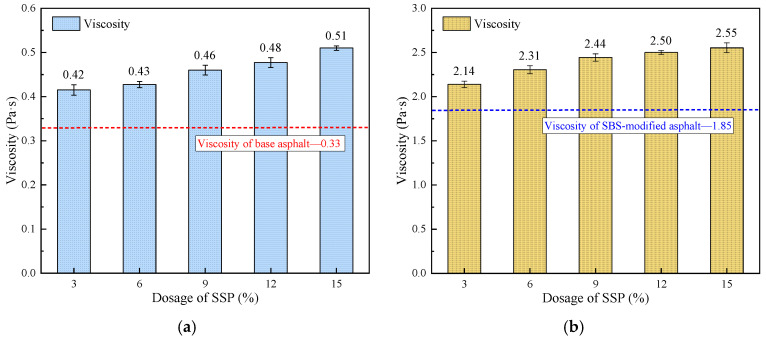
Rotational viscosity at 135 °C of base and SBS-modified asphalt with different SSP contents. (**a**) Base asphalt; (**b**) SBS-modified asphalt.

With the increasing content of SSP in base asphalt and SBS-modified asphalt, the variations in rotational viscosity at 135 °C are shown in Figure 8a,b. The rotational viscosity at 135 °C gradually increases with the increase in the SSP content, and the increase in rotational viscosity at 135 °C of base asphalt with the addition of 3%, 6%, 9%, 12%, and 15% SSP is 27.3%, 30.3%, 39.4%, 45.5%, and 54.5%, respectively. While the increase in rotational viscosity at 135 °C of SBS-modified asphalt with the same SSP content is 15.7%, 24.9%, 31.9%, 35.1%, and 37.8%, respectively. By combining with asphalt and interacting, the SSP can enhance the shear resistance of asphalt. Due to the single component and relatively simple structure of base asphalt, the improvement value in rotational viscosity at 135 °C of the base asphalt by adding SSP is relatively small. However, the increase range in the rotational viscosity at 135 °C of SBS-modified asphalt with the addition of SSP is relatively small because of the spatial three-dimensional structure of SBS-modified asphalt.

The consistency, temperature sensitivity, and shear deformation resistance of base asphalt and SBS-modified asphalt can be improved significantly according to the addition of SSP, but excessive SSP may reduce the thermal stability of asphalt. The ductility and fatigue cracking resistance of asphalt is significantly decreased after the content of SSP exceeded 9% and 12% in the base asphalt and SBS-modified asphalt, respectively.

### 3.3. High-Temperature Rheological Properties

Dynamic shear rheological tests were conducted on base asphalt and SBS-modified asphalt with SSP contents of 3%, 6%, 9%, 12%, and 15%. The rutting factor (G*/sinδ) obtained from temperature sweep tests is closely related to the high-temperature rheological properties of asphalt, and the greater the rutting factor, the more pronounced the resistance of asphalt to flow deformation. The elastic recovery ability and resistance to permanent deformation of asphalt can be evaluated with the strain recovery rate (R) and non-recoverable creep compliance (J_nr_) obtained from MSCR tests. The proportion of elastic strain in the total strain is increased with the increase of R and the decrease of J_nr_, indicating stronger elasticity and deformation resistance of the asphalt.

#### 3.3.1. Rutting Parameter

**Figure 9:**
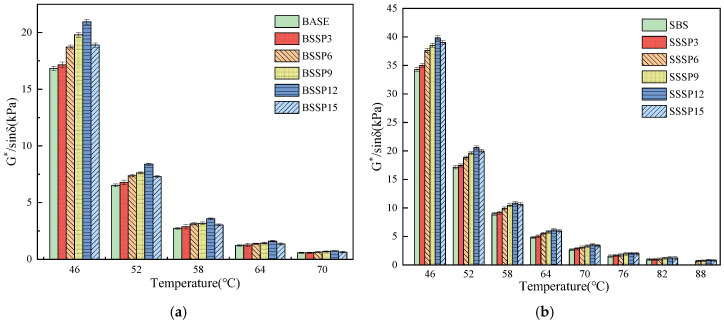
Rutting factor of base and SBS-modified asphalt with different SSP contents. (**a**) Base asphalt; (**b**) SBS-modified asphalt.

The temperature sweep test results of the rutting factor G*/sinδ of base asphalt and SBS-modified asphalt with different SSP contents are shown in Figure 9a,b.

For the same SSP content, the G*/sinδ of both base asphalt and SBS-modified asphalt is decreased with the increasing temperature, indicating that the rutting resistance of asphalt is reduced, possibly due to the increase in molecular movement of the asphalt is exacerbated by the increase in temperature. For the spatial three-dimensional structure of SBS-modified asphalt, the failure temperature of SBS-modified asphalt is significantly higher than that of base asphalt [45]. At the same temperature, the G*/sinδ of asphalt shows a trend of first increasing and then decreasing with the increase in SSP content, reaching the highest point at an SSP content of 12%. The results are consistent with the results of the softening point test mentioned earlier. When the SSP content is greater than 6%, the failure temperature level of SBS-modified asphalt is increased from 82 °C to 88 °C with an SSP content greater than 6%. This indicates that the thermal stability of asphalt is enhanced with the addition of SSP. However, because of excessive SSP on the overall structural stability of asphalt, the thermal stability of the asphalt may be adversely affected.

#### 3.3.2. Creep and Recovery Performance

**Figure 10:**
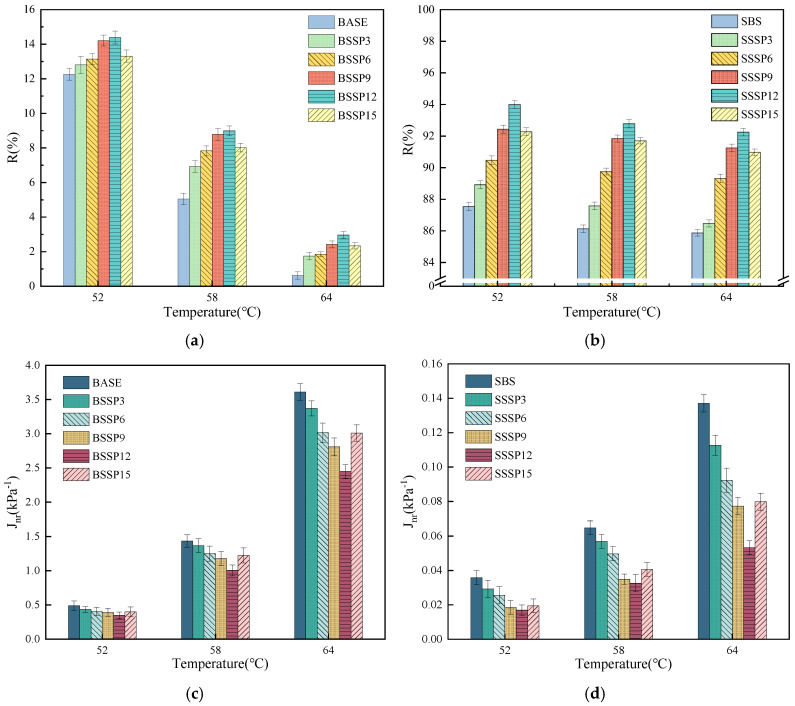
MSCR test results of base and SBS-modified asphalt with different SSP contents at the load level of 0.1 kPa. (**a**) R of base asphalt; (**b**) R of SBS-modified asphalt; (**c**) J_nr_ of base asphalt; (**d**) J_nr_ of SBS-modified asphalt.

**Figure 11:**
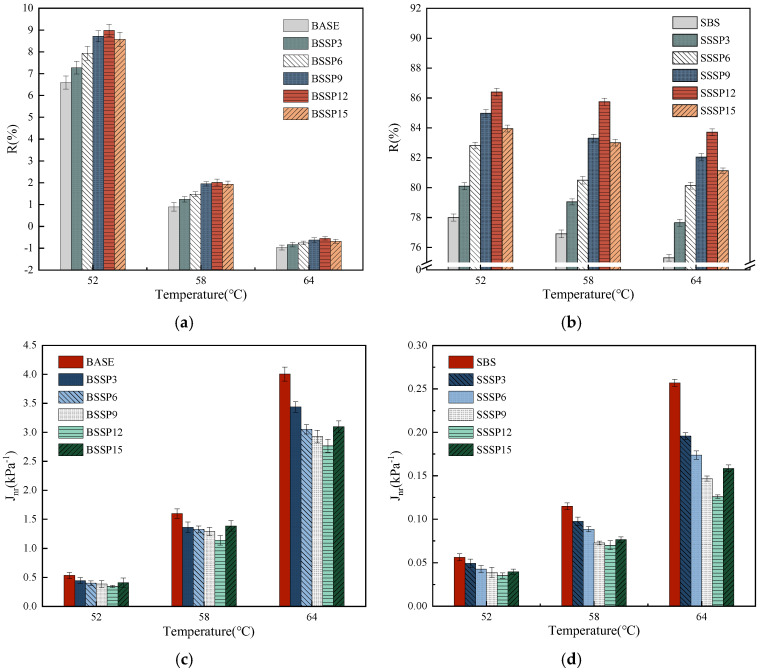
MSCR test results of base and SBS-modified asphalt with different SSP contents at the load level of 3.2 kPa. (**a**) R of base asphalt; (**b**) R of SBS-modified asphalt; (**c**) J_nr_ of base asphalt; (**d**) J_nr_ of SBS-modified asphalt.

The MSCR test results of the R and J_nr_ of the base asphalt and SBS-modified asphalt with different SSP contents at 0.1 kPa and 3.2 kPa are shown in Figure 10 and Figure 11.

At the load level of 0.1 kPa, the R of asphalt at the same temperature increases first and then decreases with increasing SSP content, while the J_nr_ shows the opposite trend with an inflection point at 12%. For example, the increase range in R of the base asphalt at 58 °C with 3%, 6%, 9%, 12%, and 15% SSP is 37.0%, 55.1%, 73.9%, 78.0%, and 58.8%, respectively, indicating that an adequate quantity of SSP can significantly enhance the strain recovery ability of the base asphalt with a single component and simple structure. However, with the increase in the content of SSP, the enhancement rate gradually slows down and even decreases. Thus, the excessive addition of SSP with a larger stiffness may reduce the uniformity of the base asphalt. However, due to the good elastic deformation ability of SBS-modified asphalt compared to the base asphalt, the range of increase in R by SSP is relatively small.

At the load level of 3.2 kPa, the change trend of R and J_nr_ of asphalt at the same temperature with increasing SSP content is basically consistent with that at the load level of 0.1 kPa. For example, the decreased degree in J_nr_ of SBS-modified asphalt at 64 °C with 3%, 6%, 9%, 12%, and 15% SSP is 17.8%, 32.7%, 43.6%, 61.1%, and 41.8%, respectively, meaning that an appropriate quantity of SSP can significantly improve the resistance to permanent deformation of SBS-modified asphalt. Under the same temperature and SSP content, the R of the base asphalt and SBS-modified asphalt clearly decreases, while J_nr_ increases significantly at the load level of 3.2 kPa compared to that at the load level of 0.1 kPa. It can be observed that the thermal stability of asphalt under a large, repeated load is weakened, and the permanent deformation is increased. With the same load level and SSP content, the R of the base asphalt and SBS-modified asphalt decreases with increasing temperature, while J_nr_ shows the opposite trend. It means that the proportion of recoverable strain in total strain and the permanent deformation resistance of asphalt is reduced with the increase in temperature.

The thermal stability and permanent deformation resistance of base asphalt and SBS-modified asphalt can be improved with the addition of appropriate SSP. Based on the fact that SSP with a rich porous structure can fully contact with asphalt, the high-temperature rheological properties of asphalt are enhanced by the molecular interaction force between uniform distributed SSP and asphalt and the strength of SSP itself collectively. However, if the content of SSP exceeds 12%, the improvement effect on the high-temperature rheological properties of asphalt begins to decrease. The reason may be that the uniformity and stability of the overall structure are affected by the excessive SSP agglomerated in asphalt. Lv et al. also found a negative effect on high-temperature rheological properties when solid waste powder was excessively added to asphalt as a modifier [32].

### 3.4. Analysis of Scanning Electron Microscope Images

**Figure 12:**
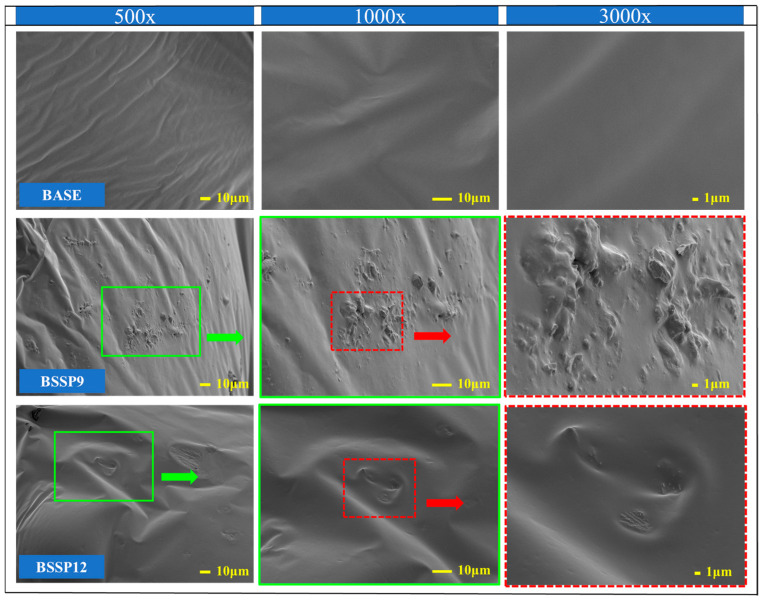
SEM images of base asphalt, BSSP9, and BSSP12.

**Figure 13:**
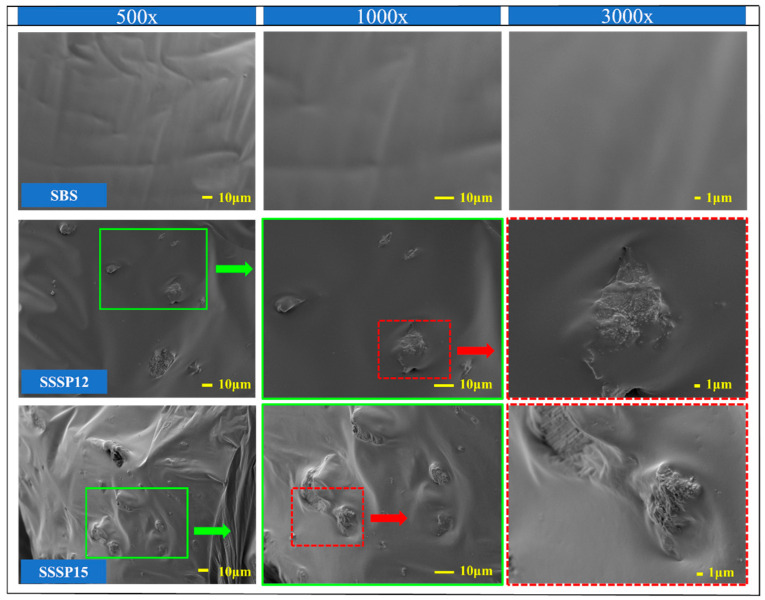
SEM images of SBS-modified asphalt, SSSP12, and SSSP15.

Based on the analysis of the results of the ductility test in Section 3.2, it was observed that the ductility of the base asphalt and SBS-modified asphalt began to decrease significantly with the content of SSP at 9% and 12%, respectively. Therefore, SEM images of the base asphalt, BSSP9, BSSP12, SBS-modified asphalt, SSSP12, and SSSP15 samples were taken at 500×, 1000×, and 3000× magnification to observe the microstructure of the SSP in the asphalt, as shown in Figure 12 and Figure 13.

At different magnifications, the surfaces of the base asphalt and SBS-modified asphalt are smooth and uniform. In the SEM images of the BSSP9 and SSSP12, it is observed that the SSP tightly combined with both types of asphalt and fully contacted, forming a stable and integrated structure with the asphalt. The above results are consistent with the improvement effect of SSP on the thermal stability of asphalt mentioned earlier. Compared to BSSP9, the distribution of SSP in SSSP12 is more uniform. The reason may be that a more complete combination and greater constraint with SSP is allowed with the higher viscosity and stable spatial network structure of SBS-modified asphalt. However, the simple overall structure of the base asphalt leads to less constraint for SSP, affecting the distribution uniformity of SSP. In the images of the BSSP12 and SSSP15, surface depressions in the samples caused by the aggregation of SSP can be observed, which are weak points that can affect the ductility of the asphalt. Because of the stronger ability to overcome the influence of agglomerated SSP with the stable internal structure of the SBS-modified asphalt, the depressions caused by the aggregation of SSP in SSSP15 are more obvious than those in BSSP12. The analysis of SEM images corresponds to the results of the ductility test above.

### 3.5. Analysis of Fourier Transform Infrared Spectroscopy

**Figure 14:**
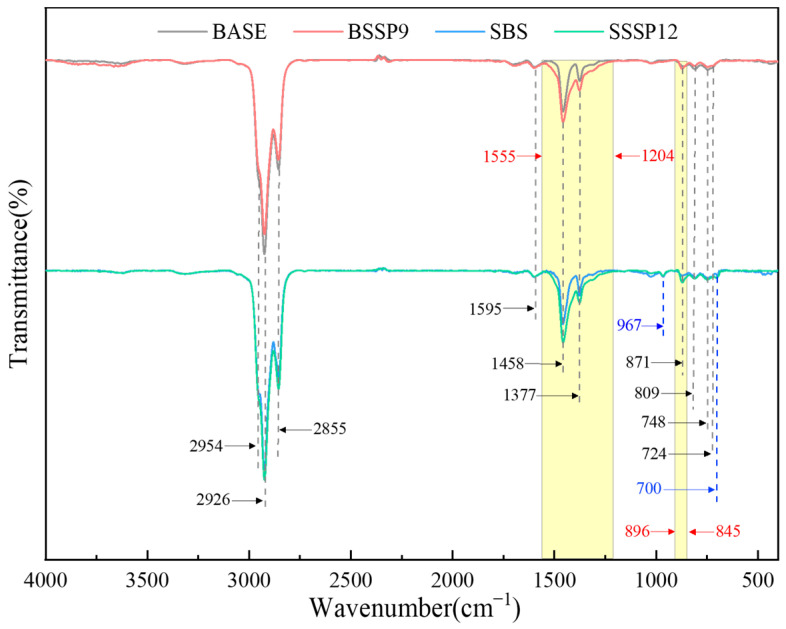
Fourier transform infrared spectra of base asphalt, BSSP9, SBS-modified asphalt, and SSSP12. However, based on the influence of the vibration of CO_3_^2−^ in SSP, the peak areas of BSSP9 and SSSP12 increase in the range of 845–896 cm^−1^ and 1204–1555 cm^−1^, indicating that the addition of SSP mainly causes physical changes in the asphalt.

In order to analyze whether a chemical reaction occurs between SSP and asphalt, four testing samples of asphalt including base asphalt, BSSP9, SBS-modified asphalt, and SSSP12 were selected for infrared spectroscopy analysis. The Fourier transform infrared spectra of the above samples are shown in Figure 14.

The positions and intensities of the absorption peak of base asphalt and SBS-modified asphalt are quite similar. The insignificant absorption peak around 2954 cm^−1^ is assigned to the -CH_3_ antisymmetric stretching vibration. Two strong absorption peaks at 2926 cm^−1^ and 2855 cm^−1^ correspond to the antisymmetric and symmetric stretching vibrations of -CH_2_-, respectively [46]. The absorption peak around 1595 cm^−1^ is attributed to the stretching vibration of C=C, and the angular vibration plane of C-CH_3_ and the bending vibration of -CH_2_- are demonstrated by the absorption peak around 1458 cm^−1^ [47]. The absorption peak at 1377 cm^−1^ is caused by the bending vibration of -CH_3_ [48]. The absorption peaks around 871 cm^−1^, 809 cm^−1^, and 748 cm^−1^ are related to the bending vibration of the benzene ring [49], and the absorption peak around 724 cm^−1^ is due to the branch chain vibration of (CH_2_)*_n_* group (*n* > 4) [50]. Compared with base asphalt, the additional absorption peaks around 967 cm^−1^ and 700 cm^−1^ of SBS-modified asphalt are, respectively, caused by the out-of-plane bending vibration of -CH_2_- in the polybutadiene block and C-H in the polystyrene block [51]. With the addition of SSP, no significant new absorption peak appears in the base asphalt and SBS-modified asphalt. However, based on the influence of the vibration of CO_3_^2−^ in SSP, the peak areas of BSSP9 and SSSP12 increase in the range of 845–896 cm^−1^ and 1204–1555 cm^−1^, indicating that the addition of SSP mainly causes physical changes in the asphalt.

## 4. Conclusions

By adding different contents of SSP to the base asphalt and SBS-modified asphalt separately, SSP-modified asphalt was prepared and tested to analyze the improvement effect of SSP on the high temperature performance of base asphalt and SBS-modified asphalt. Based on the analysis of conventional properties tests, dynamic shear rheology tests, SEM tests, and FTIR tests, the following conclusions were obtained:

(1) According to adding an appropriate amount of SSP, the viscosity, shear deformation resistance, thermal stability, and resistance to permanent deformation of asphalt can be improved. However, the high-temperature sensitivity would be reduced with excessive SSP, and the influence on the performance of base asphalt is greater than that of SBS-modified asphalt.
(2) SSP is distributed more uniformly in SBS-modified asphalt than in base asphalt because SSP can be constrained with a more stable internal spatial structure formed between SBS and asphalt. The ability of SBS-modified asphalt to resist the agglomerated SSP, a weakness, is stronger than that of base asphalt. To ensure the basic ductility and fatigue cracking resistance of asphalt, the recommended contents of SSP in base asphalt and SBS-modified asphalt are 9% and 12%, respectively.
(3) Based on the addition of SSP in the base asphalt and SBS-modified asphalt, the area of the characteristic peak caused by CO_3_^2−^ is increased without the appearance of new infrared absorption peaks. The high temperature performance of asphalt is mainly enhanced by SSP through physical modification.
(4) In order to further promote the application of SSP in asphalt, pre-treatment of SSP and optimization of the preparation conditions for different asphalts present as possible methods. The negative effect on fatigue cracking resistance and the concern about the long-term structural stability of SSP particle void structure are still to be solved for the application of SSP.

## Figures and Tables

**Table 1:** Technical specifications of base asphalt.

Test Items	Penetration (25 °C) (0.1 mm)	Softening Point (°C)	Ductility (10 °C, 5 cm/min) (cm)
Technical indicators (Grade A)	60–80	≥45	≥15
Base asphalt	75.8	47.9	36.1

**Table 2:** Technical specifications of SBS-modified asphalt.

Test Items	Penetration (25 °C) (0.1 mm)	Softening Point (°C)	Ductility (5 °C, 5 cm/min) (cm)
Technical indicators (I-C)	60–80	≥55	≥30
SBS-modified asphalt	64.10	77.7	58.6

## Data Availability

The data presented in this study are available on request from the corresponding author. The data are not publicly available due to the data involved some research secrets.
